# Applications of AI in Predicting Drug Responses for Type 2 Diabetes

**DOI:** 10.2196/66831

**Published:** 2025-03-27

**Authors:** Shilpa Garg, Robert Kitchen, Ramneek Gupta, Ewan Pearson

**Affiliations:** 1 Diabetes Endocrinology and Reproductive Biology, School of Medicine, University of Dundee, Ninewells Avenue, Dundee, DD1 9SY, United Kingdom, 44 7443787733; 2 Novo Nordisk, Oxford, United Kingdom

**Keywords:** type 2 diabetes, artificial intelligence, machine learning, drug response, treatment response prediction, ML, AI, deep learning

## Abstract

Type 2 diabetes mellitus has seen a continuous rise in prevalence in recent years, and a similar trend has been observed in the increased availability of glucose-lowering drugs. There is a need to understand the variation in treatment response to these drugs to be able to predict people who will respond well or poorly to a drug. Electronic health records, clinical trials, and observational studies provide a huge amount of data to explore predictors of drug response. The use of artificial intelligence (AI), which includes machine learning and deep learning techniques, has the capacity to improve the prediction of treatment response in patients. AI can assist in the analysis of vast datasets to identify patterns and may provide valuable information on selecting an effective drug. Predicting an individual’s response to a drug can aid in treatment selection, optimizing therapy, exploring new therapeutic options, and personalized medicine. This viewpoint highlights the growing evidence supporting the potential of AI-based methods to predict drug response with accuracy. Furthermore, the methods highlight a trend toward using ensemble methods as preferred models in drug response prediction studies.

## Introduction

Type 2 diabetes mellitus stands as one of the most common metabolic disorders, comprising 90%‐95% of all cases of diabetes and affecting millions of people worldwide. The condition arises from 2 main factors: malfunctions in insulin secretion by pancreatic β-cells and the resistance of insulin-sensitive tissues to insulin [1]. The aim of treatment for type 2 diabetes is to maintain good blood sugar (glucose) levels, which can reduce the risk of development of complications related to diabetes, such as retinopathy, nephropathy, neuropathy, and cardiovascular diseases. Initial therapies include lifestyle changes and certain medications such as metformin and sulfonylureas. The specific drug or combination of drugs used is based on individual needs and medical history. Treatment with certain drugs may be unsuccessful depending on the physiological and pathological characteristics of individuals.

There is considerable heterogeneity among people with type 2 diabetes and their response to different drugs. The use of ineffective drugs results in the deterioration of a patient’s condition and raises health care expenses. Thus, there is a need to develop reliable drug response prediction methods to help identify the efficacy of potential treatments for an individual. The heterogeneity of disease and treatment response emphasizes the need for advanced analytical methods, such as artificial intelligence (AI), to understand complex patterns within data, identify patient subgroups with distinct characteristics and ultimately pave the way for personalized and precision medicine.

The main objective of this viewpoint is to review the literature exploring the use of AI-based techniques for predicting drug response in type 2 diabetes, as well as drawing upon other disease areas such as rheumatoid arthritis, multiple sclerosis, and cardiovascular diseases. For type 2 diabetes, AI methods can help gain insights into the determinants or predictors of drug response (age, sex, type of drug, dosage, duration, medical history, ethnicity, socioeconomics, blood biochemistry, and genetics) and identify characteristics that are responsible for poor drug response. The goal is to provide an extensive overview of the key findings, methodologies, algorithms, outcomes, and limitations identified in the reviewed studies. Through a critical evaluation, this review aims to assess the strengths and weaknesses of certain AI-based algorithms in predicting treatment response and to identify potential areas of future research.

## Understanding the Role of AI

### AI in Drug Response Prediction

AI presents a compelling solution for drug response prediction due to several key factors. Traditional approaches to determining drug response often rely on limited datasets and simpler regression models, which may overlook the complex interplay of factors influencing treatment outcomes. Furthermore, these methods focus on a narrow set of variables, potentially missing crucial insights into individual patient characteristics and treatment responses. However, with the advancement of AI, particularly machine learning (ML) algorithms, there is an opportunity to leverage vast amounts of data, including electronic health records (EHRs), genomics data and real-world patient data [2]. AI enables a more comprehensive analysis, by considering multiple variables and confounders simultaneously [3]. By examining data holistically and identifying intricate patterns across diverse sources of information, AI has the potential to increase our understanding of drug response mechanisms.

### Leveraging a Diverse Data Source

There are a lot of data types available when considering drug response. AI can potentially use all of these to enable drug response prediction. The data that can be used by AI systems for observational studies includes laboratory findings, EHRs, claims and bills, genome sequencing data, clinical data, disease registries, patient-reported outcomes, data from wearable devices and sensors, pharmacogenomics data, demography data, hematology, etc [4,5]. Additionally, EHR data can itself provide detailed information about a patient’s medical history, diagnoses, treatments, drug prescription records, dosage, clinical outcomes, etc. Furthermore, genetic data of patients, such as their genomic profiles can be helpful to understand individualized treatment responses. Pharmacogenomics studies can examine genetic variations and their influence on drug responses.

### AI Techniques and Their Applications

AI is a broad field comprising a wide range of technologies and techniques for building systems that can independently perform tasks associated with human intelligence. The applications of AI in health care have been used in patient data management, predictive medicine, clinical decision-making, diagnostics, and personalized medicine [6,7]. AI includes a range of methods, among which ML and deep learning (DL) stand out as 2 prominent subsets [8]. ML is involved in building systems that are capable of learning from data, identifying patterns, and making decisions. On the other hand, DL, is a special form of ML inspired by the structure and function of the brain, especially neural networks. These models learn from data autonomously and are adaptable to various features.

The most prominent methods for prediction modelling are ensemble-based methods, such as random forest (RF) and gradient boosting machines [9-11]. These methods combine the predictions of multiple models to produce a stronger overall prediction. They can reduce overfitting and increase robustness by using the diversity of the constituent models. This is achieved by training multiple base learners on different subsets of the data or with different algorithms and then combining their predictions [12].

### Explainable Artificial Intelligence

It is important to understand how AI functions to ensure trust and transparency. This is where explainable artificial intelligence (XAI) methods come into play [13-15]. In their review, Loh et al [13] discuss XAI and its practical applications. XAI methods have undergone significant advancements to enhance our trust in a model’s predictions by providing insights into the reasoning behind them. Further, XAI proves to be a valuable tool alongside traditional statistical approaches when analyzing the connections between variables and outcomes. Some of the most popular XAI methods include local interpretable model-agnostic explanations, gradient-weighted class activation mapping, and Shapley additive explanations [16,17]. These methods are combined with ML models to make predictions. They showcase the importance of features independently of the model’s structure, and the direction of influence from predictive variables.

### Advanced Modeling Techniques

Methods exploring interactions among input variables should also be considered in predictive modelling. These techniques capture complex relationships and nonlinear effects between predictors, improving model performance. Several methods can identify potential interactions, such as introducing polynomial features, adding interaction terms by multiplying variables, using tree-based algorithms, performing feature engineering, implementing neural networks to automatically learn complex interactions, and using domain knowledge. By accounting for these interactions, predictive models can become more accurate and informative, enabling better decision-making and personalized treatment strategies.

### Ensuring Transparency and Reproducibility

In drug response studies, mainly those leveraging AI techniques, adherence to transparent and standardized reporting guidelines is important. The Transparent Reporting of a Multivariable Prediction Model for Individual Prognosis or Diagnosis guidelines [18] ensures the robustness and reliability of predictive models. These guidelines provide a structured framework for model development, validation, and performance evaluation, thus enhancing transparency and reproducibility. Moreover, adherence to TRIPOD(Transparent Reporting of a Multivariable Prediction Model for Individual Prognosis or Diagnosis) guidelines enhances the clinical relevance of predictive models by promoting clarity and consistency in reporting key elements such as patient’s characteristics, predictor variables, outcome measures, and model performance metrics.

### Model Selection and Performance Evaluation

Selecting the best AI model is a critical task. The ideal model is expected to be accurate and suitable for a specific task. Opting for a model with higher performance ensures reliable outcomes, improved predictions, and informed decision-making. Thus, performance comparison of different models is necessary to find the model with the highest accuracy and efficiency. The process involves evaluating the model’s performance against each other using a set of metrices and techniques. Performance comparison can be done through various approaches, such as root-mean-square-error, accuracy, sensitivity, specificity, precision, area under the curve (AUC), mean absolute relative difference, receiver operating characteristic curve, mean squared error, etc [9,19]. These metrices offer insights into various aspects of model performance. In terms of AUC in drug response prediction, a higher AUC indicates better discriminative ability of the model, with values closer to 1 indicating stronger predictive performance. However, the interpretation of AUC should also consider factors such as the balance between specificity and sensitivity, as well as the clinical significance of false positives and false negatives [20].

Additionally, techniques such as cross-validation can be used to obtain robust performance comparison by assessing the model’s generalization capabilities. This involves splitting the data into multiple folds and training or testing the models on subsets of data to perform a more comprehensive evaluation. It helps to reduce the chances of overfitting or underfitting by providing a more realistic estimate of the performance of any model. Methods for addressing generalizability in predictive modelling also include techniques such as bootstrapping and external validation. These methods ensure that the model’s performance is not overly influenced by the specific characteristics of the training dataset and can be applied to new populations.

## Modeling Drug Response Using AI

To better understand the key aspects of drug response prediction methods using AI-based models, we examined the existing literature on the recent ML and DL-based models in specific disease domains. A comprehensive search was conducted in July 2023, across multiple academic databases, including PubMed, Scopus, and bioRxiv, using keywords related to drug treatment response, ML, and specific disease areas. The search strategy included keywords grouped into 2 sets: “AI-based keywords” and “drug response-based keywords.” These keywords were selected based on a combination of domain knowledge, a review of existing literature, and consultation with subject matter experts. These 2 sets were combined using the Boolean operator “AND” to narrow down the search and identify relevant studies.

Keywords for AI were combined using the Boolean operator “OR” to capture a wide range of AI-related concepts: (“machine learning” OR “artificial intelligence” OR “deep learning” OR “prediction model” OR “statistical model” OR “neural network” OR “data science” OR “computational intelligence” OR “graph data” OR “machine intelligence” OR “convolutional network” OR “random forest” OR “reinforcement learning”).

Keywords for drug response were similarly combined using the Boolean operators “OR” to encompass various related terms: (“treatment response” OR “drug response” OR “response prediction” OR “treatment prediction” OR “treatment outcome” OR “drug response prediction” OR “clinical outcome” OR “therapeutic outcome”).

**Figure 1.**
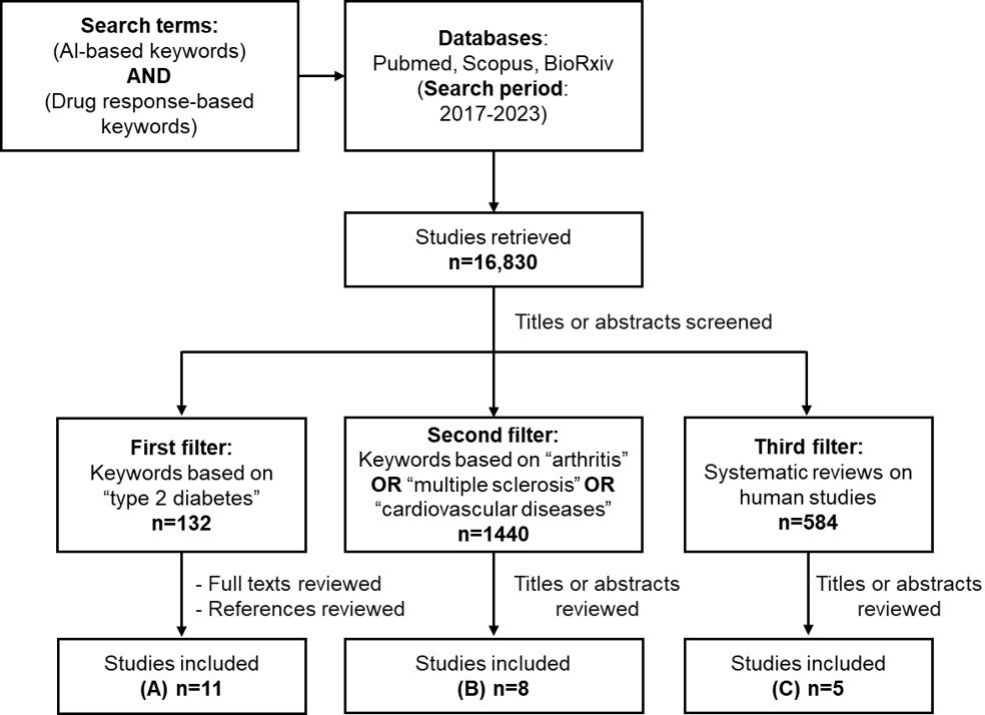
A flowchart representing this study’s selection process. (A) references [21-31], (B) references [32-39], and (C) references [5-8,13].

The studies were first filtered for “type 2 diabetes” and then for other disease areas such as arthritis, multiple sclerosis, and cardiovascular diseases (Figure 1A-C). These additional conditions were chosen because they are widely studied in relation to drug response and represent areas where AI methods have shown emerging applications. Additionally, we filtered for systematic reviews published on human studies to identify already published papers, as they provide a comprehensive summary of existing evidence.

The references of the retrieved studies were also reviewed to locate additional relevant papers. For this review, studies published between 2017 and 2023 were considered. We focused on papers that applied ML and DL algorithms specifically predicting treatment responses in clinical trials or observational studies.

### AI and Drug Response in Type 2 Diabetes

While much literature has been published on AI methods, their applications in life sciences are still comparatively limited. The field that has been most explored is oncology, where drug response prediction models are built using pharmacogenomic databases and cancer cell lines due to the impracticality and cost of clinical trials studies across diverse cancers [4,40,41]. Cardiometabolic diseases are a young upcoming field in the application of AI methodologies, likely due to limitations in data availability. The 11 studies identified in type 2 diabetes from the years 2017 to 2023 highlight the promise of data-driven insights in this field.

Most studies focus on predicting treatment responses to combinations of drugs, which aligns more closely with real-world scenarios where patients often receive multiple medications to treat the medical conditions. These studies use various criteria to make binary classification models. Some aim to predict whether a patient achieves a target HbA_1c_ (glycated hemoglobin) goal, while others focus on predicting if the patient experiences a reduction in HbA_1c_ by a certain number of units. Performance is evaluated using metrics such as AUC or accuracy, depending on the context. Additionally, we compare the quantity and nature of data used, as well as AI methods and outcomes.

In the field of drug response studies, traditional linear and logistic regression models have been staples for quite some time. For instance, Pantalone et al [21] developed a logistic regression model on 6973 patients to predict responders—patients who achieve an HbA_1c_ goal of less than 8% when treated with a combination of multiple antidiabetic drugs (Table 1). Their binary classification model achieved an AUC of 0.648. In a separate observational study, Wang et al [22] used a logistic regression model alongside multiple ML models on 2787 patients’ data to predict patients who achieve an HbA_1c_ goal of less than 7% when treated with insulin. While the logistic regression model yielded an accuracy of 0.55, the RF reached an accuracy of 0.75, and both the back propagation artificial neural network and the support vector machine achieved an accuracy of 0.73. Notably, the support vector machine, RF, and back propagation artificial neural network models outperformed the logistic regression model in the accuracy metric. Both studies relied on traditional logistic regression models, which, as indicated by the results, demonstrated lower performance compared to ML methods [21,22]. These traditional models assume linear relationships between variables, which may not be well-suited for real-world data. As a result, they fail to capture the necessary associations for making accurate predictions.

**Table 1.** Studies incorporating AI^a^ to predict treatment response in type 2 diabetes using clinical trials or observational data.

Reference	Study objective	Data type and number of patients (n)	Drug treatment (single or in combination)	AI methods	Prediction outcome	Performance
Tao et al [27]	Machine learning models to predict fasting blood glucose and HbA_1c_^b^ after 3 months of treatment	Retrospective study n=2169	Metformin, sulfonylurea, thiazolidinediones, GLP-1^c^, DPP-4^d^, SGLT2^e^, acarbose, meglitinide, insulin	Logistic regression, SGD^f^, decision tree, Gaussian NB^g^, QDA^h^, Bernoulli NB, LDA^i^, Multinomial NB, RF^j^, Extra Tree, passive aggressive, AdaBoost, begging, GBM^k^, XGBoost, ensemble learning	Reach HbA_1c_ target below 7%	AUC^l^ (ensemble)>0.9
Berchialla et al [24]	Machine learning models to predict treatment outcome	Clinical trials n=385	Metformin, sulfonylurea, DPP-4 inhibitors	Ensemble algorithm (super learner: GBM, GLM^m^, RF, MARS^n^, SVM^o^, CART^p^, BART)^q^	Reduction in HbA_1c_ of at least 0.5%	AUC: 0.92
Sun et al [28]	Effective treatment recommendations using reinforcement learning	Observational study n=189,520	Metformin, sulfonylurea, thiazolidinediones, DPP-4, GLP-1, SGLT2, acarbose (AGI^r^), basal insulin, premixed insulin	Multivariate logistic regression, reinforcement learning	Odds of achieving target HbA_1c_<7% among concordant compared to nonconcordant group	Odds ratio: 1.73 (95% CI 1.69 to 1.76)
Pantalone et al [21]	Prediction model on probability of HbA_1c_ goal attainment	Retrospective cohort study n=6973	Metformin, sulfonylurea, thiazolidinediones, DPP-4, GLP-1, SGLT2, AGI, insulin	Logistic regression	Reach HbA_1c_ target below 8%	AUC: 0.648 (95% CI 0.633 to 0.663)
Wang et al [22]	Machine learning models for predicting HbA_1c_ among patients treated with insulin	Observational study n=2787	Insulin	Logistic Regression, RF, SVM, BP-ANN^s^	Reach HbA_1c_ target below 7%	AUC (LR^t^): 0.74 AUC (RF): 0.75 AUC (SVM): 0.72 AUC (BP-ANN): 0.72
Dennis [29]	Using individualized prediction models to optimize selection of treatment	Observational study n=8798	Metformin, sulfonylurea, thiazolidinediones, DPP-4, GLP-1, SGLT2	Individualized prediction models	3-year change from baseline in HbA_1c_	Reduction in HbA_1c_ (mmol/mol): Concordant: −16.9 (95% CI −18.2 to ‐15.6)
Lopez et al [25]	Predicting the response to short-term intensive insulin therapy	Clinical trial n=24	Insulin	RF	Percentage change in ISSI-2^u^	AUC: 0.951
Ngufor et al [30]	Mixed effect machine learning for predicting longitudinal change in HbA_1c_	Observational study n=27,005	Metformin, sulfonylurea, thiazolidinediones, insulin, Meglitinide, AGI, GLP-1, DPP-4, amylinomimetics	Mixed effect Machine learning, RF, GBM, GLMs	Reach HbA_1c_ target below 7%	AUC: 0.7‐0.8
Del Parigi et al [23]	Machine learning to identify predictors of drug response	Phase III clinical trial data n=1363	SGLT2, DPP-4	RF, classification trees	Reach HbA_1c_ target below 7%	Prediction accuracy: 0.77‐0.82
Nagaraj et al [31]	Machine learning models to predict short and long-term HbA_1c_ response	Observational study n=1188	Insulin	Generalized linear regression, SVM, RF	Reduction in HbA_1c_≥5 mmol/mol or reach target HbA_1c_ below ≤53 mmol/mol	AUC (short term): 0.80 (95% CI 0.78 to 0.83) AUC (long term): 0.81 (95% CI 0.79 to 0.84)
Murphree et al [26]	Machine learning models to predict response after 1 year of metformin therapy	Health records n=12,147	Metformin	Stacked classifiers (ensemble): LR, RF, NN^v^, k-NN^w^, stochastic gradient boosting, SVM, CART, averaged neural network, FDA^x^, GBM, PLS^y^, SLDA^z^	Reach HbA_1c_ target below 7%	AUC: 0.58‐0.75

a AI: artificial intelligence.

b HbA_1c_: glycated hemoglobin.

c GLP-1: glucagon-like peptide 1.

d DPP-4: dipeptidyl peptidase 4.

e SGLT2: sodium-glucose cotransporter 2.

f SGD: stochastic gradient descent.

g NB: Naïve Bayes.

h QDA: quadratic discriminant analysis.

i LDA: linear discriminant analysis.

j RF: random forest.

k GBM: gradient boosted machine.

l AUC: area under the curve.

m GLM: generalized linear model.

n MARS: multivariate adaptive regression spline.

o SVM: support vector machine.

p CART: classification and regression tree.

q BART: Bayesian additive regression tree.

r AGI: alpha-glucosidase inhibitor.

s BP-ANN: back propagation artificial neural network.

t LR: linear regression.

u ISSI-2: insulin secretion-sensitivity index-2.

v NN: neural network.

w k-NN: k-nearest neighbor.

x FDA: flexible discriminant analysis.

y PLS: partial least square.

z SLDA: sparse linear discriminant analysis.

Some of these studies use clinical trial data, which is more organized, and cleaner compared to observational data for building ML models. Del Parigi et al [23] used a clinical trial data of 1363 patients and applied 2 ML algorithms, namely RF and classification trees, to find predictors of glycemic control in patients treated with a combination of sodium-glucose cotransporter 2 and dipeptidyl peptidase 4 inhibitors, both as dual-therapy and mono-therapy. The prediction accuracy of their models ranged from 0.77 to 0.82, with fasting plasma glucose and HbA_1c_ emerging as the most influential predictors of achieving glycemic control.

Berchialla et al [24] used a clinical trial data of 385 patients and used a weighted combination of 7 algorithms (Table 1) using an ensemble approach known as the super learner to predict responders, specifically patients who achieve a reduction in HbA_1c_ of at least 0.5% when treated with conventional drugs and dipeptidyl peptidase 4 inhibitors. Their ensemble model yielded an AUC of 0.92. In a different study, Lopez et al [25] used clinical trial data from 24 patients to develop an RF model for predicting the response to short-term intensive insulin therapy. Their binary classification model yielded an accuracy of 0.91 and an AUC of 0.951. These 2 analyses yield very high AUC values, which raise some concerns. Their sample sizes are very small, presenting a high risk of overfitting. Models trained on such limited data may not generalize well to broader populations. Additionally, with a small sample size, there is a higher risk of selection bias, where the characteristics of the patients could be very similar and may not represent larger populations. This can skew the results and lead to an overestimation of model performance.

We found that most studies that used ML approaches used ensemble-based methods to build predictive models [22-27,30]. Ensemble-based techniques, such as gradient boosting machines, RFs, and stacking, have become popular due to their high performance and capability to work with complex datasets. For instance, Murphree et al [26] established an ensemble-based ML model using 20 base models (Table 1) to predict glycemic response after 1 year of metformin therapy. Their models achieved AUC values ranging from 0.58 to 0.75 with baseline HbA_1c_, metformin dosage, and diabetic complications being the strongest predictors. In a different study, Tao et al [27], also developed ensemble-based ML models to predict patients who achieve an HbA_1c_ goal of less than 7% after 3 months of treatment with multiple antidiabetic drugs. They compared the performance of 16 different ML models (Table 1), where AUC values of the top 5 models were all greater than 0.9. Overall, these ensemble-based methods have the capability to combine multiple weak learners and generate a more accurate and robust final model, that can reduce bias and overfitting, resulting in better predictions [42,43]. Additionally, these methods have become more accessible with the development of user-friendly libraries and packages, which helps researchers use them effectively.

All these ML models identified the significant features associated with drug response. The most crucial indicators of drug response included the patient’s baseline HbA_1c_, fasting blood glucose, BMI, medication compliance, dietary habits, age, race, family history, diabetes duration, blood pressure, and dosage and usage of specific antidiabetic drugs [21-31]. These variables are derived from a combination of clinical trials and health records.

These studies provide a basis for understanding observational data, clinical data, interpreting drug responses, using statistical and ML algorithms, and suggesting tools and packages for data analysis. In most of the studies, a general trend of using ensemble-based models is observed, but it is essential to consider other DL-based modelling techniques for more complex datasets or when dealing with nonlinear relationships between variables. These advanced AI methods can offer the potential to find predictive factors that can help identify patients who can benefit most from a given treatment.

### AI and Drug Response in Other Disease Areas

Exploring disease areas other than diabetes that have used ML models for predicting drug responses can offer a broader perspective and valuable insights. By studying how AI models are applied in other disease contexts, we can adapt and refine these methods for type 2 diabetes. Further, learning additional techniques for data processing, feature engineering, and cross-validation can enhance the reliability of AI-driven drug response models. We identified numerous examples in the literature of the application of ML and DL methodologies in various disease domains [5,32-39], including rheumatoid arthritis, multiple sclerosis, cardiovascular disorders, and neurological conditions (Table 2).

**Table 2.** Studies incorporating AI^a^ to predict treatment response using clinical trials or observational data in nondiabetes conditions.

Reference	Study objective	Disease state	Data type and number of patients (N)	AI methods	Performance
Zhao et al [36]	Machine learning and statistical analysis to predict drug treatment outcome	Pediatric epilepsy	Retrospective study n=103	Multilayer perceptron, logistic regression, Naïve Bayes, SVM^b^, RF^c^, decision tree	AUC:^d^ 0.812
Duong et al [37]	Using machine learning to find clinical predictors of drug response	Rheumatoid arthritis	Clinical trial data n=775	LASSO^e^ regression, RF	AUC (LASSO): 0.74‐0.84 AUC (RF): 0.62‐0.73
Myasoedova et al [38]	Using machine learning for individualized prediction of drug response	Rheumatoid arthritis	Observational study n=643	RF	AUC: 0.84
Falet et al [35]	Using deep learning to estimate individual treatment effect on disability progression	Multiple sclerosis	Clinical trial data n=3830	Multilayer perceptron	HR:^f^ 0.743
Koo et al [32]	To develop machine learning models for predicting remission in patients treated with biologics.	Rheumatoid arthritis	Observational study n=1204	LASSO and ridge regression, SVM, RF, XGBoost, SHAP^g^	Accuracy: 52.8%‐72.9% AUC: 0.511‐0.694
Liang et al [39]	Machine learning to predict response after cardiac resynchronization therapy	Cardiovascular disease	Retrospective study n=752	LR^h^, SVM, RF, LASSO, ridge, NN^i^, EN^j^, k-NN^k^, XGBoost	AUC>0.77
Norgeot et al [34]	Using longitudinal deep learning model to predict controlled or uncontrolled state with clinical disease activity index	Rheumatoid arthritis	Electronic health records n=820	Longitudinal deep learning	AUC (UH^l^ cohort): 0.86‐0.96 AUC (SNH^m^ cohort: 0.65‐0.83)
Guan et al [33]	Using AI to predict the responses to TNF^n^ inhibitors in patients using clinical and genetic markers	Rheumatoid arthritis	Observational study n=2572	Gaussian process regression model	AUC: 0.66 Correlation coefficient: 0.405

a AI: artificial intelligence.

b SVM: support vector machine.

c RF: random forest.

d AUC: area under the curve.

e LASSO: least absolute shrinkage and selection operator.

f HR: hazard ratio.

g SHAP: Shapley additive explanation.

h LR: linear regression.

i NN: neural network.

j EN: elastic net.

k k-NN: k-nearest neighbor.

l UH: university hospital.

m SNH: safety-net hospital.

n TNF: tumor necrosis factor.

In the case of rheumatoid arthritis, Koo et al [32] developed multiple ML models (Table 2) for prediction of remission in patients who are treated with biologic disease-modifying antirheumatic drugs. They used Shapley additive explanation values for explaining the predictions and ranking of important features. The AUC for these models ranged from 0.511 to 0.694. Guan et al [33] developed a Gaussian process regression model for the prediction of responses in terms of changes in Disease Activity Score-28 to tumor necrosis factor inhibitors. They used clinical and genetics data, and their model yielded an AUC of 0.66. In another study, Norgeot et al [34] developed a longitudinal DL model with clinical disease activity index to predict controlled (low activity or remission) or uncontrolled state (moderate or high activity). The AUC ranged from 0.86 to 0.96 in 1 cohort and from 0.65 to 0.83 in another cohort.

For predicting treatment response to anti-CD20 monoclonal antibodies in multiple sclerosis, Falet et al [35] used a DL-based method called multilayer perceptron (MLP). Their model yielded hazard ratio of 0.743. Similarly, Zhao et al [36] used multiple ML models (Table 2) and MLP in case of pediatric epilepsy to predict the drug treatment outcomes of antiseizure medications. Their top performing MLP model achieved an AUC of 0.812. The MLP is based on a neural network architecture with the ability to approximate any mathematical function, handle nonlinear relationships and work with diverse datasets. MLPs can compute outputs based on input data through a process called feed propagation. MLPs use an optimization algorithm called backpropagation to adjust the weights and minimize the prediction error. The flexibility of MLPs contribute to their role in various classification and regression tasks [44,45].

## Challenges and Limitations

### Data Quality and Accessibility

Using AI for predicting treatment response from observational studies comes with several challenges and limitations that must be carefully considered. First, obtaining high-quality and diverse patient data, including longitudinal and genetic data, can be challenging. Obtaining individual-level patient data linked to health outcomes can be restricted in several geographic regions, and not adequately linked. Real-world data often presents a high burden of curation and contains gaps, such as mixed-up units or incorrect health care recordings which diminish the data quality. Moreover, there are very few data sources that offer harmonized data across different medical systems, further complicating analysis, and interpretation.

### Data Biases and Missingness

Limited or biased data may prevent the AI model’s ability to make precise predictions across various patient populations. Biases in the data could arise from various sources, such as demographic biases (eg, underrepresentation of certain age groups or ethnicities), clinical biases (eg, overrepresentation of patients with certain medical conditions or treatments), or geographic biases (eg, data collected predominantly from specific regions or health care settings). Furthermore, data limitations could arise from insufficient sample sizes, imbalanced class distributions, missing or incomplete data points, etc. These limitations can impact a model’s ability to perform better.

It is also possible that some of the important predictive factors are not measured and therefore not included in most of the analyses. For instance, when predicting disease progression or treatment response, factors such as patient’s socioeconomic status, medication history, adherence to treatment regimens, genetic variations, or lifestyle behaviors (eg, diet or exercise) could be critical for accurate predictions. However, if these factors are not routinely collected or integrated into the analysis, the model’s predictive performance may be compromised.

### Data Security and Privacy

It is important to address concerns related to data security and privacy when handling patient data. Health care organizations must safeguard sensitive patient information from unauthorized access or misuse to ensure patient confidentiality. Additionally, there are ethical considerations in AI pertaining to how AI systems are developed, deployed, and used in health care. AI models should not discriminate against certain demographic groups or perpetuate existing biases in health care delivery.

### Model Interpretability, Validation, and Clinical Integration

Furthermore, ensuring the interpretability and explainability of AI models is crucial, as clinicians and researchers require insight into the factors influencing predictions for improved understanding and translation, to see increased adoption. Thorough validation and testing of the model’s performance on an independent patient set is essential to ensure the clinical utility. Moreover, the integration of AI models into existing clinical workflows requires clinical collaborations. Addressing these challenges requires a collective action from stakeholders across the health care ecosystem, including researchers, policy makers, health care providers, and technology developers. By acknowledging and overcoming these challenges, AI can be a valuable tool in predicting treatment responses.

## Conclusion

This viewpoint highlights the potential of AI in predicting treatment response in people with type 2 diabetes as well as other diseases. From this literature survey, we discovered that methods such as Gaussian process regression and DL techniques such as the MLP that have been used successfully in other disease areas have not been extensively investigated for predicting drug responses in type 2 diabetes. Yet, they show significant potential for developing prediction models due to several factors. Gaussian process regression offers the advantage of providing probabilistic predictions, which can capture uncertainty in the data. On the other hand, DL techniques such as the MLP has capabilities to learn complex patterns and representations from large-scale datasets, which is useful in capturing heterogeneous drug response.

After reviewing the literature, it becomes evident that integrating diverse data sources, using feature selection algorithms, implementing effective model optimization strategies, and validation through external validation have collectively resulted in the development of robust predictive models. Moving forward, it is essential to continue exploring the innovative approaches to overcome limitations, such as the interpretability, the curse of dimensionality [46], and low-quality data.

Our viewpoint sheds light on the limitations of traditional statistical models in handling high-dimensional data effectively. To overcome these constraints, advanced ML methods should be considered, such as ensemble methods and DL, which demonstrate high performance in handling complex datasets. However, while these models excel in predictive accuracy, their opaque nature presents challenges in understanding the contributions of individual features to predictions. This underscores the importance of exploring methods to enhance the transparency and interpretability of models by including XAI techniques.

In summary, the literature reviewed demonstrates the successful use of AI methods for predicting drug responses in type 2 diabetes, while also identifying key clinical predictors of drug response. These models lay the foundation for the development of treatment recommendation systems, offering the potential for enhanced diabetes management, and ultimately leading to improved patient care.

## Abbreviations

AI: artificial intelligence
AUC: area under the curve
DL: deep learning
EHR: electronic health record
HbA_1c_: glycated hemoglobin
ML: machine learning
MLP: multilayer perceptron
RF: random forest
XAI: explainable artificial intelligence
